# Left Atrial Myxoma With Pleural Effusion

**DOI:** 10.4021/jocmr2009.11.1269

**Published:** 2009-11-24

**Authors:** Mehmet Akif Cakar, Caner Arslan, Ahmet Yildiz, Mehmet Bulent Vatan, Huseyin Gunduz

**Affiliations:** a Sakarya Education and Research Hospital, Cardiology Department, Turkey; b 29 May Private Hospital, Cardiovascular Surgery Department, Turkey; c 29 May Private Hospital, Cardiology Department, Turkey

## Abstract

Cardiac myxomas are histologically benign tumors but they may be lethal because of their strategic position. They have variable presentations and excellent prognosis after surgical excision. Left atrial myxomas may mimic mitral stenosis and is very rarely associated with pleural effusion. We presented a case of left atrial myxoma blocking mitral orifice and causing recurrent pulmonary edema and pleural effusions in this report.

**Keywords:**

Left Atrial; Myxoma; Pleural effusion

## Introduction

Myxomas are the most common primary cardiac tumors. They are usually benign and occur more frequently in women. These are mostly located in the left atrium and uncommonly in the right atrium. Sudden death may occur in patients with atrial myxoma due to tumor embolization or obstruction of blood flow at mitral or tricuspid valve. Approximately 50% of patients with myxomas may experiences symptoms due to central or peripheral embolism or intracardiac obstruction, but 10% of patients may be completely asymptomatic [1]. Detection of myxoma is an indication for urgent cardiosurgical intervention due to the rapid growth of tumor, risk of embolism, and blocking of the outflow. Cardiosurgical treatment consists of removal of the tumor with its pedicle. The association of atrial myxoma with pleural effusion appears to be rare and is usually due to left atrial obstruction [2]. In this report, we presented a case of left atrial myxoma mimicking mitral stenosis and causing bilateral pleural effusion.

## Case Report

A 67-year-old woman was admitted with a history of malaise, dyspnea and non productive cough for the 3 years to the local state hospital. She has a 15-year history of hypertension and diabetes mellitus. Two weeks prior to admission, the patient also had difficulty of breathing even on slight activity. She was hospitalized for 5 days with diagnosis of heart failure and her complaints were resolved after diuretic treatment and drainage 1.5 and 2 liters of serous fluid from the right pleura.

One week after discharge from the hospital, she was admitted to our tertiary center with the same complaints. On physical examination, the heart rate was regular at 108 beats/min and blood pressure was 100/70 mmHg and she was orthopneic. Jugular veins were distended. Respiratory sounds were not audible on the lower portions of the lung fields and there was dullness to percussion in both bases posteriorly. Fine crepitations were also heard on the mid portions of the chest bilaterally. Diastolic rumbling murmur of 2 - 3/6 in intensity without opening snap was present on the mitral valve area.

Arterial blood gas under room air conditions showed severe hypoxia with an oxygen partial of 49.0 mmHg and oxygen saturation of 81%. A complete blood count showed a low hemoglobin level of 10.8 gr/dl and hematocrit value of 32.8%. Leukocyte count was slightly higher than normal (12.3 x 10^3^/mm^3^). Serum cross-reacting protein (CRP) and erythrocyte sedimentation rate (ESR) were increased to 22.2 mg/dl and 101 mm/hr, respectively. All other routine laboratory data were within normal limits.

**Figure 1:**
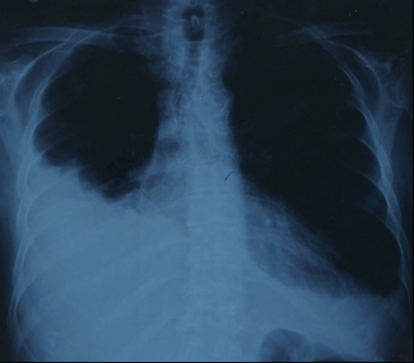
Chest X-ray shows bilateral pleural effusion.

**Figure 2:**
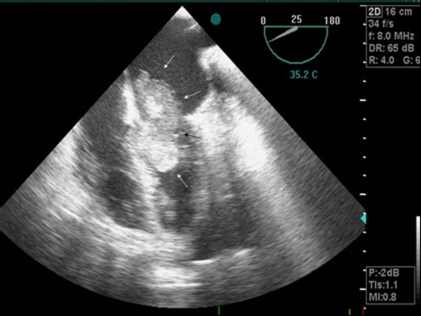
Left atrial myxoma projection into the left ventricle during diastole.

Chest radiography revealed moderate cardiomegaly and bilateral pleural effusion which was prominent on the right side (Fig. 1). Electrocardiography demonstrated sinus tachycardia. Transesophageal echocardiography (TEE) confirmed the diagnosis of the left atrial mass previously described in TTE (Fig. 2). There was a normal thickness and contractility of the left ventricular walls with an ejection fraction rate of 65%. Coronary angiography ruled out coronary artery disease, and catheterization was not performed to avoid tumor embolization.

**Figure 3:**
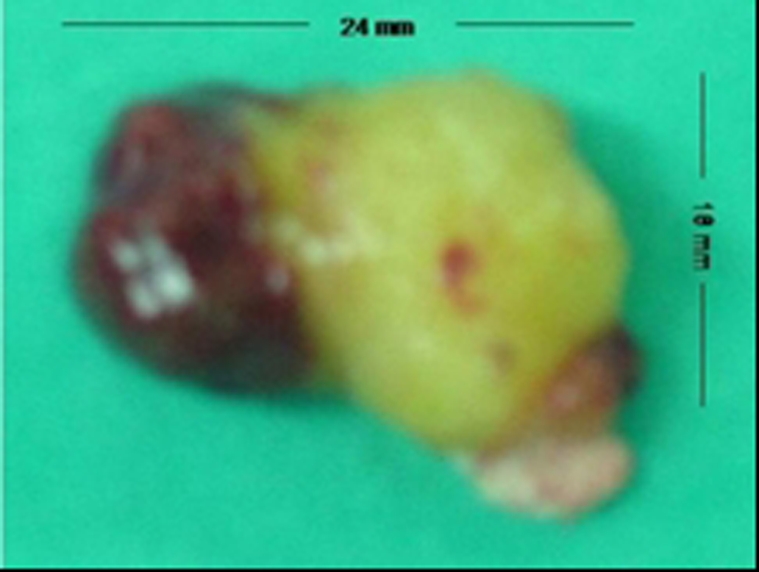
Left atrial polypoid myxoma extirpated with atrial septum.

Then, the patient was scheduled for emergency operation. After cardiopulmonary bypass was instituted and right atrium was opened, interatrial septum was opened around the tumor dimple with a nearly 9 - 10 mm normal septal tissue cuff, and myxomatous tumor mass of 4.5 x 2.3 x 2.1 cm in size was totally extirpated with its pedicle without any fragmentation (Fig. 3).

The left atrium, left ventricle, right atrium and right ventricle were inspected for evidence of concomitant tumors. After mitral valve was controlled for any insufficiency, the defect in the atrial septum was closed with fresh pericardium. Operation was ended after TEE confirmation of only a minimal mitral valve insufficiency. Pathological investigation of the tumor material verified the diagnosis of myxoma (spindle shaped and elongated cells with round and ovoid nuclei and prominent nucleoli embedded in a myxomatous stroma).

The patient's postoperative course was unremarkable and she was discharged home on postoperative day 5. Leukocytosis, elevated CRP, ESR returned to value within a month of surgery and she had no pleural effusion. She remained completely asymptomatic 16 months after surgery with no evidence of recurring tumor and is being followed under simple antihypertansive therapy.

## Discussion

Although rare, atrial myxomas constitute the most common benign cardiac tumors with an incidence of up to 3 in 1000 patients. They most frequently present to cardiologist with signs and symptoms of mitral valve disease or with embolic phenomena [1]. Clinical symptoms of cardiac myxomas are often very confusing. The most common symptom associated with cardiac myxoma is congestive heart failure, followed by either systemic or pulmonary embolization [3]. However, the patients may rarely present with symptoms mimicking mitral valve obstruction and pleural effusion. Cases of right [4] and left [5] pleural effusion suggesting heart failure [6] or systemic disease [7] were occasionally reported in the literature. In patient with myxoma and pleural effusion recurring postoperative period was also rarely reported [8]. It has been thought that pleural effusion is caused by the increase in left atrial pressure because of the mitral valve obstruction. Over the following days to weeks, additional fluid may accumulate due to the deposition of excess pleural space. In our case, we have thought that the cause of pleural effusion is same obstructive mechanism.

Recent progress in diagnostic modalities including echocardiography, computed tomography and magnetic resonance imaging, has allowed a definitive diagnosis of primary cardiac tumors without the need for cardiac catheterization that were hazardous for potential embolization in patients with known myxomas. Likewise, we early detected the tumor in the present case with TTE. In the majority of cases, the diagnosis of myxoma is made during echocardiographic examination for differential diagnosis of dyspnea, syncope, cardiac murmur or an embolic event. With the advent of more accurate imaging techniques, myxoma incidence in the elderly patients has been noted to be higher than originally described. In patient with mitral valve stenosis, however, chronic atrial thrombus remains an important differential diagnosis of myxoma. TTE is approximately 95% sensitive for detection of cardiac myxomas and TEE approaches 100% sensitivity [9].

Early diagnosis and surgical removal can lead to rapid resolution of symptoms with avoidance of devastating complications like peripheral embolism and valve dysfunction. Excision of atrial myxomas using cardiopulmonary bypass has been established with generally good clinical results. However, one must use an individualized approach based on the location of tumors to control possible embolization. Transseptal approach was preferred in the present case since it allows total resection of the left atrial myxoma [10].

It is also important to note that serious mitral insufficiency may be overlooked during preoperative investigations, as the tumor mass may block mitral valve orifice. The control of the mitral valve for insufficiency should be necessary after tumor removal to repair the valve. As a result, this case has shown that early two-dimensional echocardiography is advisable as possible myxoma especially for pleural effusion in patients with heart failure.
